# Empagliflozin for Heart Failure With Preserved Left Ventricular Ejection Fraction With and Without Diabetes

**DOI:** 10.1161/CIRCULATIONAHA.122.059785

**Published:** 2022-06-28

**Authors:** Gerasimos Filippatos, Javed Butler, Dimitrios Farmakis, Faiez Zannad, Anne Pernille Ofstad, João Pedro Ferreira, Jennifer B. Green, Julio Rosenstock, Sven Schnaidt, Martina Brueckmann, Stuart J. Pocock, Milton Packer, Stefan D. Anker

**Affiliations:** National and Kapodistrian University of Athens School of Medicine, Athens University Hospital Attikon, Greece (G.F.).; Baylor Scott and White Research Institute, Dallas, TX (J.B.).; Department of Medicine, University of Mississippi School of Medicine, Jackson (J.B.).; University of Cyprus Medical School, Nicosia (D.F.).; Centre d’Investigations Cliniques Plurithématique 14-33, Inserm U1116, CHRU, F-CRIN INI-CRCT (Cardiovascular and Renal Clinical Trialists), Université de Lorraine, Nancy, France (F.Z., J.P.F.).; Medical Department, Boehringer Ingelheim Norway KS, Asker (A.P.O.).; Oslo Diabetes Research Center, Norway (A.P.O.).; Cardiovascular Research and Development Center, Faculty of Medicine of the University of Porto, Portugal (J.P.F.).; Duke Clinical Research Institute, Duke University School of Medicine, Durham, NC (J.B.G.).; Dallas Diabetes Research Center at Medical City, TX (J.R.).; Boehringer Ingelheim Pharma GmbH & Co KG, Biberach, Germany (S.S.).; Boehringer Ingelheim International GmbH, Ingelheim, Germany (M.B.).; First Department of Medicine, Faculty of Medicine Mannheim, University of Heidelberg, Germany (M.B.).; Department of Medical Statistics, London School of Hygiene & Tropical Medicine, UK (S.J.P.).; Baylor University Medical Center, Dallas, TX (M.P.).; Imperial College, London, UK (M.P.).; Department of Cardiology and Berlin Institute of Health Center for Regenerative Therapies, German Centre for Cardiovascular Research (DZHK) partner site Berlin, Charité Universitätsmedizin, Berlin, Germany (S.D.A.).; Institute of Heart Diseases, Wrocław Medical University, Poland (S.D.A.).

**Keywords:** death, diabetes mellitus, heart failure, hospitalization, prognosis

## Abstract

Empagliflozin improves outcomes in patients with heart failure with a preserved ejection fraction, but whether the effects are consistent in patients with and without diabetes remains to be elucidated.

**Methods:**

Patients with class II through IV heart failure and a left ventricular ejection fraction >40% were randomized to receive empagliflozin 10 mg or placebo in addition to usual therapy. We undertook a prespecified analysis comparing the effects of empagliflozin versus placebo in patients with and without diabetes.

**Results:**

Of the 5988 patients enrolled, 2938 (49%) had diabetes. The risk of the primary outcome (first hospitalization for heart failure or cardiovascular death), total hospitalizations for heart failure, and estimated glomerular filtration rate decline was higher in patients with diabetes. Empagliflozin reduced the rate of the primary outcome irrespective of diabetes status (hazard ratio, 0.79 [95% CI, 0.67, 0.94] for patients with diabetes versus hazard ratio, 0.78 [95% CI, 0.64, 0.95] in patients without diabetes; *P*_interaction_=0.92). The effect of empagliflozin to reduce total hospitalizations for heart failure was also consistent in patients with and without diabetes. The effect of empagliflozin to attenuate estimated glomerular filtration rate decline during double-blind treatment was also present in patients with and without diabetes, although more pronounced in patients with diabetes (1.77 in diabetes versus 0.98 mL/min/1.73m^2^ in patients without diabetes; *P*_interaction_=0.01). Across these 3 end points, the effect of empagliflozin did not differ in patients with prediabetes or normoglycemia (33% and 18% of the patient population, respectively). When investigated as a continuous variable, baseline hemoglobin A1c did not modify the effects on the primary outcome (*P*_interaction_=0.26). There was no increased risk of hypoglycemic events in either subgroup as compared with placebo.

**Conclusions:**

In patients with heart failure and a preserved ejection fraction enrolled in the EMPEROR-Preserved (Empagliflozin Outcome Trial in Patients With Chronic Heart Failure With Preserved Ejection Fraction), empagliflozin significantly reduced the risk of heart failure outcomes irrespective of diabetes status at baseline.

**Registration:**

URL: https://www.clinicaltrials.gov; Unique identifier: NCT03057951.

**Clinical Perspective**

**What Is New?**

- In the placebo-controlled EMPEROR-Preserved trial (Empagliflozin Outcome Trial in Patients With Chronic Heart Failure With Preserved Ejection Fraction), empagliflozin improved heart failure outcomes and slowed kidney function decline in patients with heart failure and a preserved ejection fraction regardless of the presence of diabetes at baseline.
- In patients without diabetes, empagliflozin did not lower glycated hemoglobin or increase the risk of hypoglycemia or ketoacidosis.

**What Are the Clinical Implications?**

- The heart failure benefits, slowing of kidney function decline, and safety of empagliflozin in patients with heart failure and preserved ejection fraction are consistent in patients with and without diabetes.
- Decisions regarding the use of empagliflozin for the treatment of heart failure with preserved ejection fraction should not be driven by the glycemic status of individual patients.

Diabetes is present in nearly half of patients with heart failure with preserved left ventricular ejection fraction (HFpEF) and is associated with increased morbidity and mortality.^1,2^ An analysis of the TOPCAT trial (Treatment of Preserved Cardiac Function Heart Failure With an Aldosterone Antagonist) suggested that patients with diabetes were clustered within a phenotype characterized by a specific profile of comorbidities such as obesity and chronic kidney disease, along with more advanced symptoms and worse outcomes.^3^ Sodium–glucose cotransporter-2 inhibitors (SGLT2i) have been shown to improve outcomes in patients with diabetes; a meta-analysis including data from 5 SGLT2i trials in nearly 50000 patients with diabetes showed that SGLT2i reduced the risk of the composite of cardiovascular death/myocardial infarction/stroke, as well as the risk of cardiovascular death, heart failure (HF) hospitalization, and renal events.^3^ In addition, the seminal studies on SGLT2i in HF with reduced ejection fraction have shown that SGLT2i improve outcomes in these patients independently of diabetes status.^4,5^ Whether this benefit is maintained in patients with diabetes and HF and preserved or mildly reduced ejection fraction has not been sufficiently studied.

The treatment of patients with HFpEF has long been characterized by lack of therapies that improve prognosis. Empagliflozin, an SGLT2i, reduced the risk of the composite of cardiovascular death or HF hospitalization and first and recurrent HF hospitalizations and slowed renal function decline in patients with HF and an ejection fraction >40% in EMPEROR-Preserved (Empagliflozin Outcome Trial in Patients With Chronic Heart Failure With Preserved Ejection Fraction).^6^ We sought to assess the effects of empagliflozin in these patients according to their diabetes status at baseline.

## Methods

### Study Design

EMPEROR-Preserved was an international, phase III, double-blinded, parallel-group, placebo-controlled trial that enrolled 5988 patients with symptomatic HF, an ejection fraction >40%, elevated natriuretic peptide levels, and evidence of structural cardiac changes or documented previous hospitalization for HF. Patients were randomized to empagliflozin 10 mg daily or placebo. The primary end point was the time to first hospitalization for HF or cardiovascular death. The key secondary end points included first and recurrent hospitalizations for HF and the rate of decline in the estimated glomerular filtration rate (eGFR) during double-blind treatment (eGFR slope). Other secondary end points included time to cardiovascular death, first hospitalization for HF, and all-cause death and change in Kansas City Cardiomyopathy Questionnaire (KCCQ) clinical summary score from baseline to week 52. Other prespecified end points included the mean eGFR change from baseline to 23 to 45 days after treatment discontinuation, allowing the evaluation of double-blind treatment unconfounded by the presence of an SGLT2i, as well as time to a first composite renal end point (defined as time to first occurrence of chronic dialysis, renal transplantation, sustained reduction of ≥40% in eGFR, or sustained eGFR <15 mL/min/1.73m^2^ for patients with baseline eGFR ≥30 mL/min/1.73m^2^ or <10 mL/min/1.73m^2^ for patients with baseline eGFR <30 mL/min/1.73m^2^). The trial further assessed the effects of empagliflozin versus placebo on additional clinical and laboratory outcomes. The methods of the trial are described in detail elsewhere.^7,8^ The trial was approved by the ethics committee at each study site and all patients provided written informed consent.

### Trial Outcomes

In the current secondary analysis of the trial, we assessed the effects of empagliflozin versus placebo on the primary and secondary HF and renal end points according to the diabetes status of patients at baseline. We also analyzed the effects on the primary end point across the hemoglobin A1c (HbA1c) continuum at baseline. We further assessed the effects of empagliflozin versus placebo on the change of clinical and laboratory outcomes from baseline to follow-up, including KCCQ clinical summary score, HbA1c, body weight, systolic blood pressure, NT-proBNP (N-terminal B-type natriuretic pro-peptide), hemoglobin, and uric acid, according to baseline diabetes status. In accordance with the corresponding secondary analysis of the EMPEROR-Reduced trial,^9^ diabetes was defined as a history of diabetes diagnosis or a pretreatment HbA1c of 6.5% or higher, prediabetes was defined as a pretreatment HbA1c of 5.7% to 6.4%, and normoglycemia was defined as all pretreatment HbA1c values <5.7%.

### Statistical Analysis

For time to first event analyses, differences between the placebo and empagliflozin groups for the primary end point were assessed for statistical significance using a Cox proportional hazards model, with prespecified covariates of age, sex, geographical region, diabetes status at baseline, left ventricular ejection fraction, eGFR at baseline, treatment, and diabetes status at baseline by treatment interaction term. These analyses were performed according to the intention-to-treat principle for all randomized patients and included data up to the end of the planned treatment period. For the analysis of total (first and repeated) events, between-group differences were assessed using a joint frailty model, with cardiovascular death as a competing risk. For the analysis of changes in eGFR, KCCQ scores, vital signs, and laboratory measurements, treatment effects were assessed using a mixed model for repeated measures including age, baseline eGFR (CKD-EPI formula [Chronic Kidney Disease Epidemiology Collaboration equation] on the basis of creatinine), and baseline left ventricular ejection fraction as linear covariates and baseline measurement by visit, visit by treatment by baseline diabetes status, sex, geographic region, last projected visit on the basis of dates of randomization, and trial closure. NT-proBNP was log-transformed before analysis. Between-group differences in the slope of change in eGFR were analyzed using a random intercept random slope model including baseline eGFR, age, and baseline left ventricular ejection fraction as linear covariates and region, sex, baseline eGFR by time interaction, treatment by diabetes at baseline, and time by treatment by diabetes at baseline interaction as fixed effect(s). Intercept and slope allowed us to vary randomly between patients. KCCQ as well as eGFR (including both slope and mixed model for repeated measures analyses) were analyzed using on-treatment data. For comparison of incidence rates or slope between patients with and without diabetes, similar models as described above were used, restricting the study population to the placebo group and comparing patients with and without diabetes instead of treatment group.

Analyses for safety were performed including all patients who had received at least 1 dose of empagliflozin or placebo. All analyses were performed using SAS, version 9.4 (SAS Institute). All *P* values reported are 2-sided and *P*<0.05 was considered statistically significant in all cases. No adjustments for multiple testing were made.

### Data Sharing Statement

To ensure independent interpretation of clinical study results and enable authors to fulfill their role and obligations under the International Committee of Medical Journal Editors criteria, Boehringer Ingelheim grants all external authors access to relevant clinical study data. In adherence with the Boehringer Ingelheim Policy on Transparency and Publication of Clinical Study Data, scientific and medical researchers can request access to clinical study data after publication of the primary article in a peer-reviewed journal, regulatory activities are complete, and other criteria are met. Researchers should use the https://vivli.org/ link to request access to study data and visit https://www.mystudywindow.com/msw/datasharing for further information.

## Results

### Baseline Characteristics

Out of a total of 5988 patients enrolled in the study, 2938 (49%) had diabetes at baseline, including 10 patients (0.2%; 5 patients in each treatment group) with type 1 diabetes, and the remaining with type 2 diabetes. Among 2938 patients with diabetes, the large majority had a history of diabetes; only 277 patients had newly identified diabetes on the basis of a pretreatment HbA1c value ≥6.5%. Compared with patients without diabetes, those with diabetes were younger, were more frequently men, and had higher body mass index and HbA1c levels (Table 1). Patients with diabetes also had a higher prevalence of ischemic heart disease as the principal cause of HF and more commonly a history of coronary artery disease and hypertension, but a lower prevalence of atrial fibrillation. Furthermore, patients with diabetes had a worse New York Heart Association class but lower levels of NT-proBNP than those without diabetes. Patients with diabetes had higher median urinary albumin-to-creatinine ratio with a higher prevalence of microalbuminuria and macroalbuminuria, but similar eGFR. They were more frequently treated with angiotensin receptor blockers, diuretics, mineralocorticoid receptor antagonists, aspirin, and lipid-lowering agents, and less frequently with anticoagulants. Glucose-lowering agents other than SGLT2i had been prescribed in 79.5% of patients with diabetes; the most commonly used agent was metformin, followed by insulin and sulfonylureas.

**Table 1.**
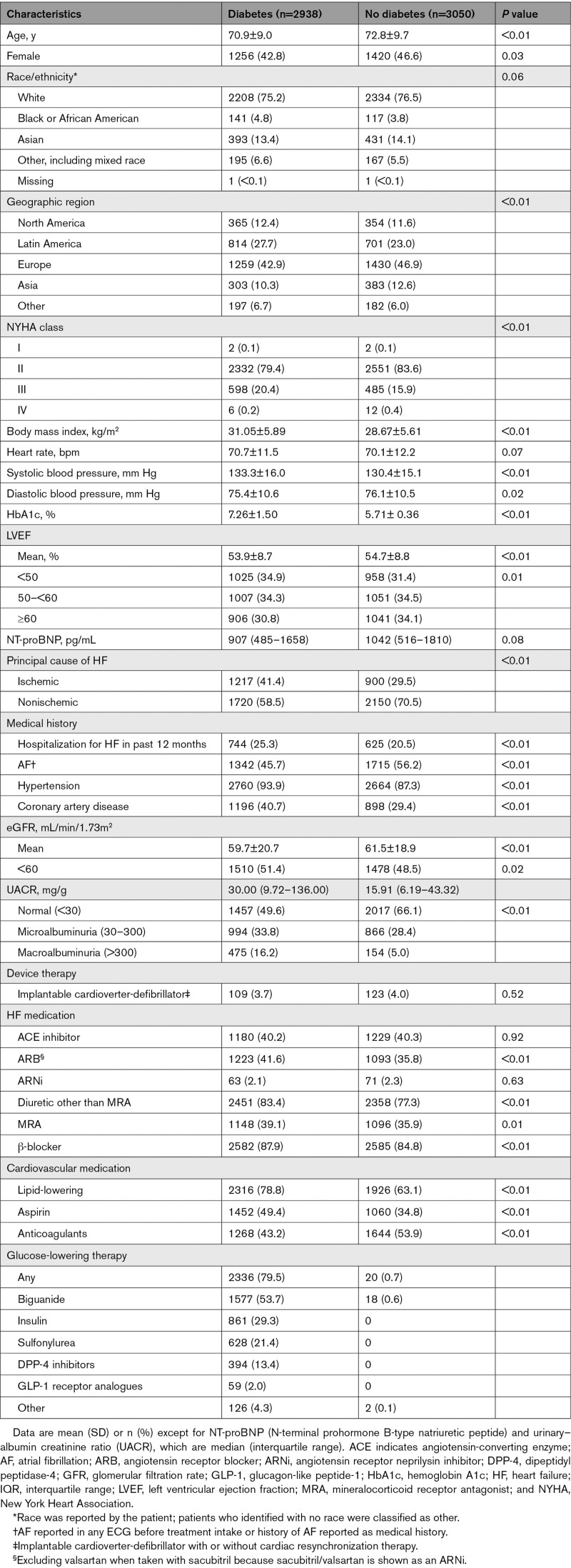
Baseline Characteristics in Patients With and Without Diabetes

Among the 3050 (51%) patients without diabetes at baseline, 1980 (33% of the total population) had prediabetes and 1070 (18% of the total population) had normoglycemia. Baseline characteristics of patients with normoglycemia, prediabetes, and diabetes are provided in Table S1.

### Heart Failure Outcomes and Health Status

**Figure 1.**
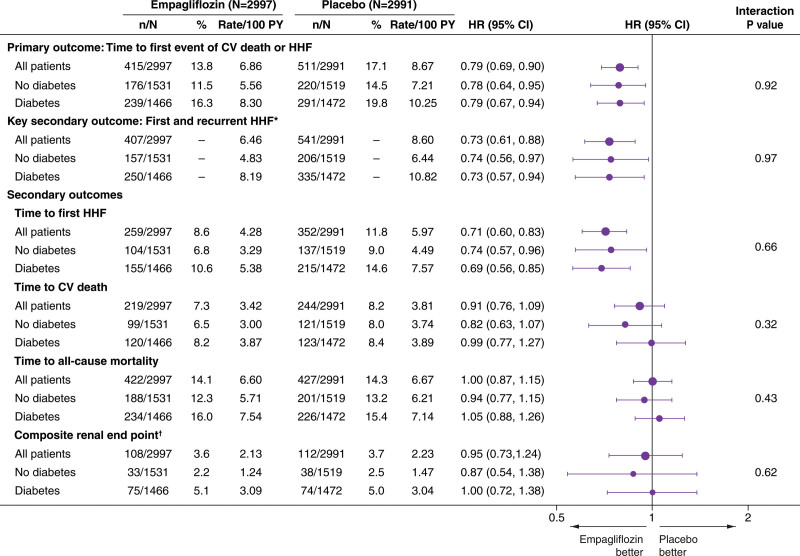
**Forest plots for the effects of empagliflozin vs placebo on the primary end point and secondary cardiovascular end points according to the presence or absence of diabetes at baseline.** n corresponds to the number of events in recurrent event analyses and the number of patients with an event for the time to first event analysis. *Recurrent event analyses are on the basis of the joint frailty model accounting for competing risk of cardiovascular death. †Time to first occurrence of (1) chronic dialysis; (2) renal transplantation; (3) sustained reduction of ≥40% in estimated glomerular filtration rate (eGFR); or (4) sustained eGFR <15 mL/min/1.73m^2^ for patients with baseline eGFR ≥30 mL/min/1.73m^2^ or <10 mL/min/1.73m^2^ for patients with baseline eGFR <30 mL/min/1.73m^2^. HHF indicates hospitalization for heart failure; HR, hazard ratio; and PY, patient-years.

In the placebo arm, the incidence rate of the primary end point, hospitalization for HF or cardiovascular death, per 100 patient-years was 10.25 in patients with diabetes versus 7.21 in those without diabetes (*P*<0.01). Similarly‚ the incidence rate of first and recurrent hospitalizations for HF per 100 patient-years was 10.82 in patients with diabetes and 6.44 in those without diabetes (*P<*0.01). Both the primary end point and first and recurrent hospitalizations for HF occurred less frequently in patients with prediabetes compared with those with diabetes, whereas the rates between the prediabetes and normoglycemic groups were similar (Figure S1). In contrast, there were no differences in the rates of cardiovascular death in the placebo group according to diabetes status (Figure 1).

**Figure 2.**
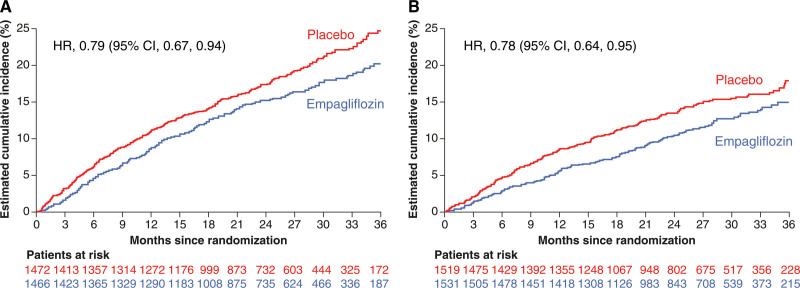
**Effect of empagliflozin vs placebo on the primary end point of cardiovascular death or first hospitalization for heart failure in patients with and without diabetes.** Effect of empagliflozin vs placebo on the primary end point of cardiovascular death or first hospitalization for heart failure in (**A**) patients with diabetes at baseline and (**B**) patients without diabetes. HR indicates hazard ratio.

**Figure 3.**
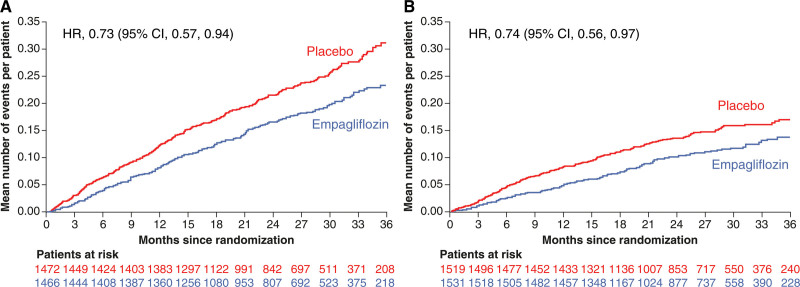
**Effect of empagliflozin vs placebo on first and recurrent hospitalizations for heart failure in patients with and without diabetes.** Effect of empagliflozin vs placebo on first and recurrent hospitalizations for heart failure in (**A**) patients with diabetes at baseline and (**B**) patients without diabetes (mean cumulative function). HR indicates hazard ratio.

**Figure 4.**
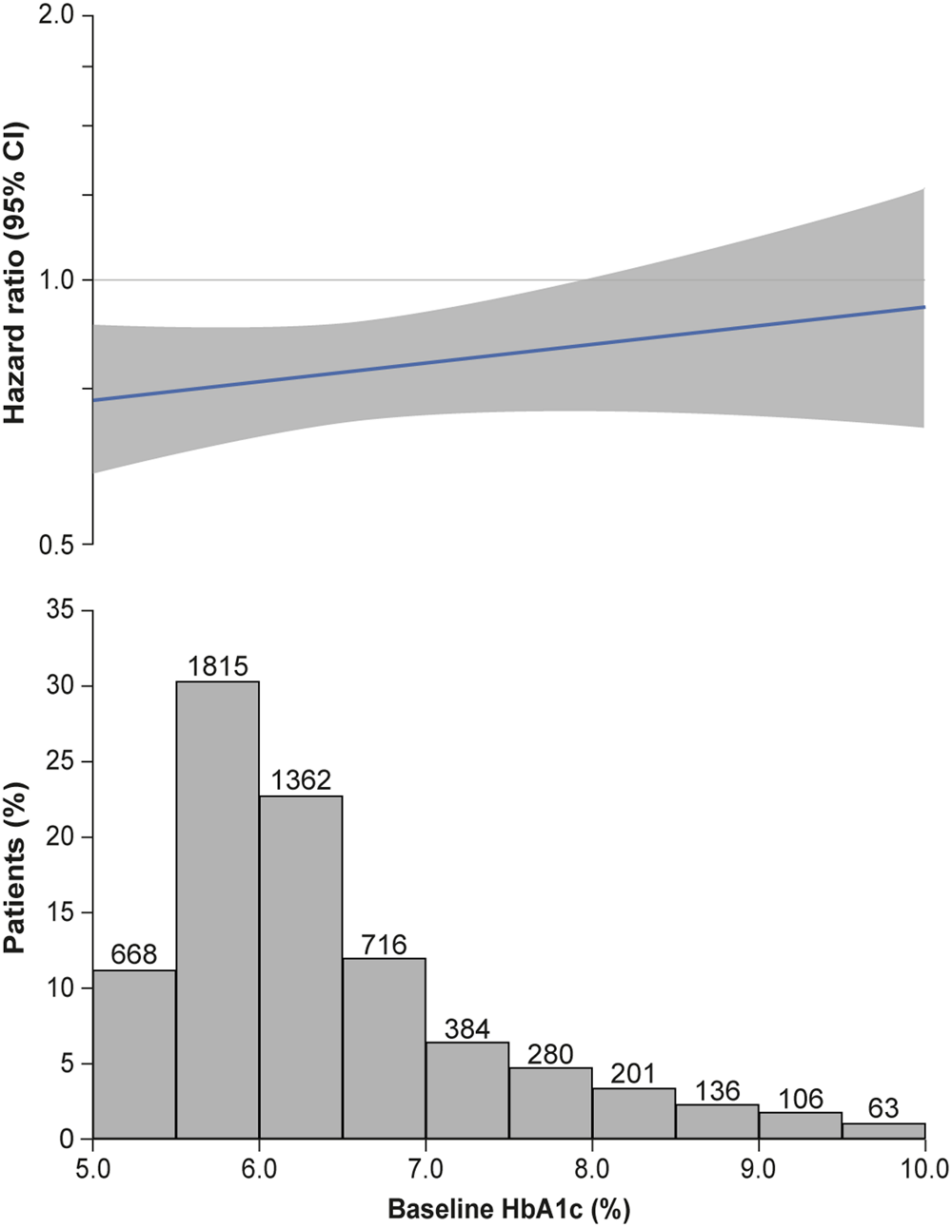
**Effect of empagliflozin on the primary end point by baseline HbA1c as a continuous variable.** The figure shows the linear association between hemoglobin A1c (HbA1c) and log hazard ratio for the primary end point. The nonsignificant interaction *P* value (0.26) indicates that the slope is not significantly different from zero. However, the display makes assumptions about the linearity that are difficult to validate, and the slope is strongly influenced by a relatively small number of patients with extreme values.

Empagliflozin reduced the risk of the primary end point of cardiovascular death or first hospitalization for HF consistently in patients with and without diabetes (*P*_interaction_=0.92; Figures 1 and 2). Similarly‚ there was no interaction between the presence of diabetes at baseline and the effects of empagliflozin versus placebo on the key secondary end point of first and recurrent hospitalizations for HF (*P*_interaction_=0.97; Figures 1 and 3) or any of the secondary HF end points including cardiovascular death, first hospitalization for HF, and all-cause death (Figure 1). This lack of interaction between baseline diabetes status and the effects of treatment with empagliflozin or placebo persisted when patients were divided into 3 subgroups of normoglycemia, prediabetes, and diabetes (Figure S1). In addition, the effect of empagliflozin on the primary outcome compared with placebo was consistent across the spectrum of HbA1c when analyzed as a continuous variable (*P*_interaction_=0.26; Figure 4). Empagliflozin further improved health status by KCCQ clinical summary score consistently in patients with and without diabetes up to week 52 (*P*_interaction_ at week 52=0.45; Figure S2).

### Renal Outcomes

In the placebo arm, patients with diabetes had a higher rate of eGFR decline than those without diabetes (adjusted on-treatment eGFR slope, −3.1 versus −2.2 mL/min/1.73m^2^ per year, respectively; *P*<0.01) and a larger decline in eGFR from baseline to 23 to 45 days after treatment discontinuation (adjusted mean eGFR change, −6.1 versus −5.3 mL/min/1.73m^2^ per year, respectively; *P*=0.02). When we further assessed the prediabetes and normoglycemic groups separately, patients with prediabetes at baseline had a higher rate of eGFR decline in the placebo group compared with those with diabetes; there was no difference between the prediabetes and normoglycemic groups (−2.1 versus −2.5 mL/min/1.73m^2^ per year, respectively; Figure S3). There were relatively few renal composite events in the placebo group (74 in patients with diabetes and 38 in those without diabetes), with higher placebo incidence rates among those with diabetes than without diabetes (3.04 versus 1.47 events per 100 patient-years, respectively; *P*<0.01).

**Figure 5.**
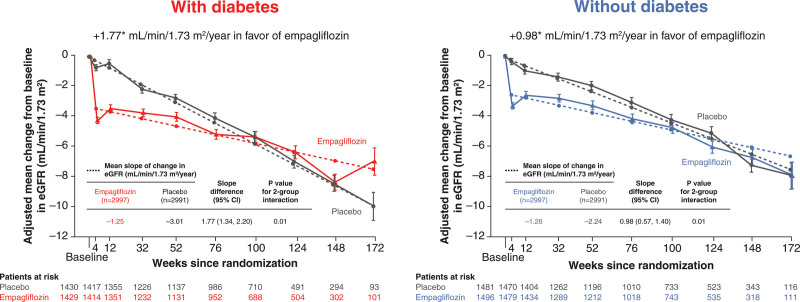
**Adjusted mean changes from baseline in eGFR (CKD-EPI) and mean slope of change in eGFR by treatment status (empagliflozin or placebo) in patients with and without diabetes at baseline.** Adjusted mean changes from baseline in estimated glomerular filtration rate (eGFR) in the 2 groups are shown as calculated with the Chronic Kidney Disease Epidemiology Collaboration equation. The bars indicate standard errors. The on-treatment data were analyzed using a mixed model for repeated measures.

Compared with placebo, empagliflozin slowed eGFR decline during study treatment in patients with and without diabetes, but with a greater magnitude in those with than in those without diabetes (adjusted slope/year difference, 1.77 [95% CI, 1.34, 2.20] versus 0.98 [95% CI, 0.57, 1.40], respectively; *P*_interaction_=0.01; Figure 5). In contrast, the effect of empagliflozin compared with placebo in reducing the mean eGFR change from baseline to follow-up 23 to 45 days after treatment discontinuation was similar in patients with and without diabetes (adjusted mean eGFR change, 2.3 mL/min/1.73m^2^ [95% CI, 1.1, 3.4] in diabetes versus 2.6 mL/min/1.73m^2^ [95% CI, 1.5, 3.6] in patients without diabetes; *P*_interaction_=0.73). When the group without diabetes was split into prediabetes and normoglycemia, the effects on eGFR slope were more pronounced in those with diabetes compared with those with prediabetes or normoglycemia, but similar in the latter two (*P*_interaction_ trend across the 3 categories=0.03; Figure S3). There was no effect on the risk of the prespecified renal composite for empagliflozin versus placebo in patients with or without diabetes observed (hazard ratio, 1.00 [95% CI, 0.72, 1.38] versus hazard ratio, 0.87 [95% CI, 0.54, 1.38], respectively; *P*_interaction_=0.62; Figure 1).

### Change in Body Weight, Blood Pressure, and Laboratory Outcomes

The effects of empagliflozin compared with placebo on the change in body weight, blood pressure, and laboratory outcomes are presented in Figures S4 and S5. HbA1c levels decreased with empagliflozin compared with placebo in patients with baseline diabetes but not in those without (*P*_interaction_ at week 52<0.01). Body weight also decreased with empagliflozin both in patients with and without diabetes, but the magnitude of this effect was generally greater in patients with diabetes (*P*_interaction_ at week 52=0.02). Hemoglobin increased with empagliflozin compared with placebo arm in patients with and without diabetes but systolic blood pressure was not notably affected in either of the 2 subgroups. There was a similar decrease in NT-proBNP levels with empagliflozin compared with placebo in patients with and without diabetes in the first weeks (*P*_interaction_ at week 12=0.30; Figure S5); thereafter, this decrease tended to be greater in patients with diabetes (*P*_interaction_ at week 52=0.03). Compared with placebo, uric acid concentration decreased in the empagliflozin arm, less profoundly in patients with diabetes than in those without diabetes up to week 100 (all *P*_interaction_<0.01; Figure S5).

### Adverse Events

The occurrence of overall adverse events and those leading to treatment discontinuation were similar in patients treated with empagliflozin and placebo but serious adverse events tended to be lower in patients on empagliflozin. These results were consistent in patients with and without diabetes (Table 2). Apart from genital infections, urinary tract infections, and volume depletion (including hypotension), which occurred more often with empagliflozin than placebo, the rates of adverse events were comparable in the treatment arms in patients with and without diabetes. Similarly‚ confirmed hypoglycemic episodes and hypoglycemic episodes requiring assistance occurred more frequently in patients with diabetes than in those without diabetes, with no difference between treatment arms (Table S2). Diabetic ketoacidosis occurred only in patients with diabetes but the rates were similar in the 2 treatment arms, with 4 (0.3%) cases in the empagliflozin group and 5 (0.3%) in the placebo group.

**Table 2.**
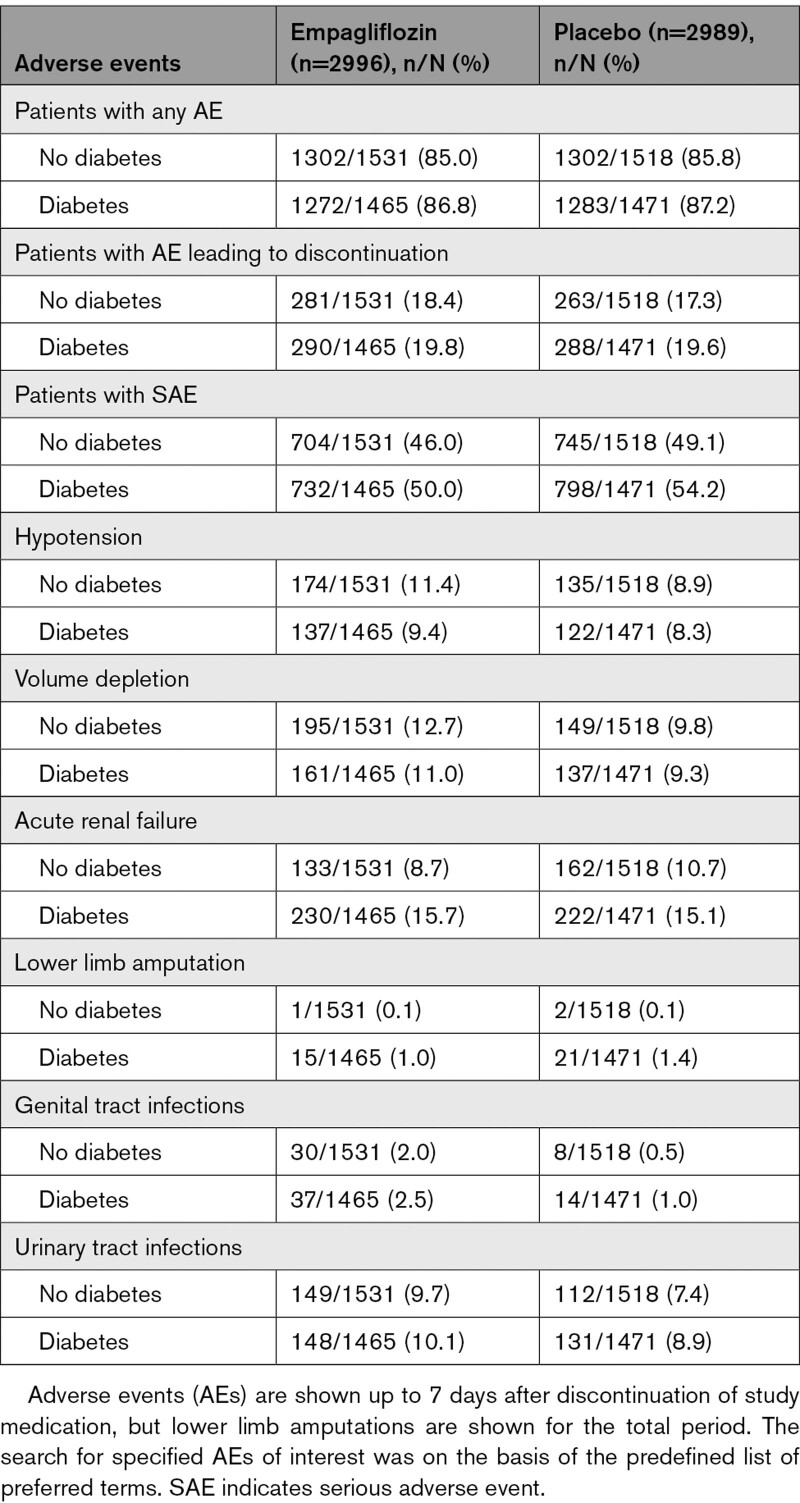
AEs and SAEs Observed in the 2 Treatment Arms According to the Presence or Absence of Diabetes at Baseline

## Discussion

In this prespecified secondary analysis of EMPEROR-Preserved in patients with symptomatic HF and an ejection fraction >40%, the effects of empagliflozin compared with placebo to reduce the rates of the primary end point of cardiovascular death or hospitalization for HF and the secondary end point of total hospitalizations for HF were consistent in patients with and without diabetes at baseline. Empagliflozin slowed eGFR decline in patients with and without diabetes but with a more pronounced effect among patients with diabetes. When the subgroup without diabetes was divided into prediabetes and normoglycemia, treatment effects remained consistent across these 2 subgroups. In addition, empagliflozin improved health status, increased hemoglobin levels, and reduced natriuretic peptides, body weight, and uric acid irrespective of baseline diabetes status, whereas it reduced HbA1c only in patients with diabetes.

Evidence from clinical trials and registries shows that diabetes is particularly prevalent in HFpEF. In the PARAGON-HF trial (Prospective Comparison of ARNI With ARB Global Outcomes in HFpEF), diabetes was present in 43% of patients at baseline.^10^ In the Get With The Guidelines HF registry, diabetes was present in 45% of patients.^11^ In accordance with this latter registry, in the current study, patients with diabetes were younger, were more frequently men, and had a higher prevalence of ischemic HF, coronary artery disease, and hypertension along with more frequent macroalbuminuria and microalbuminuria compared with those without diabetes. The fact that people with diabetes had a lower median NT-proBNP value despite their worse New York Heart Association class in the current and previous studies may be explained in part by their higher body mass index, because obesity has been associated with lower levels of natriuretic peptides.^12^ In addition, patients with diabetes studied herein also had a lower prevalence of atrial fibrillation history at baseline compared with those without diabetes, which may explain further their lower baseline NT-proBNP values. This finding is in line with previous findings: the prevalence of a history of atrial fibrillation also tended to be lower in patients with versus without diabetes in the previous HFpEF trial TOPCAT and in patients with HF with reduced ejection fraction enrolled in both EMPEROR-Reduced and DAPA-HF (Dapagliflozin and Prevention of Adverse Outcomes in Heart Failure).^9,13^

The presence of diabetes has been associated with worse outcomes in patients with HFpEF. In the TOPCAT trial, the subgroup of patients with diabetes, obesity, and chronic kidney disease had the highest rates of cardiovascular death and hospitalization for HF.^14^ In accordance with these findings, in the current study, patients with diabetes in the placebo arm had a higher incidence of HF events, including the composite of cardiovascular death or hospitalization for HF and first and recurrent hospitalizations for HF, than those without diabetes. The same was true for renal function, with a greater rate of eGFR decline in the placebo arm among patients with diabetes compared with patients without diabetes. In contrast, the rates of the primary and key secondary end points did not differ between patients with prediabetes and those with normoglycemia at baseline. Interestingly‚ these findings contrast those from PARAGON-HF, in which patients with prediabetes had an intermediate risk between that of patients with diabetes and those with normoglycemia for most HF outcomes.^15^ Our findings are nevertheless consistent with those observed in patients with HF with reduced ejection fraction enrolled in the EMPEROR-Reduced trial,^9^ which showed no difference in risk between patients with prediabetes and those with normoglycemia. Notably‚ there were no differences in the incidence of cardiovascular death across the 3 glycemia groups. Previous studies, however, including PARAGON-HF, have demonstrated that patients with diabetes have higher mortality rates compared with patients without diabetes,^16^ also in the setting of HFpEF.^15,17^

We demonstrate herein that empagliflozin reduced the risk of HF outcomes in patients with HFpEF with or without diabetes at baseline. The EMPEROR-Reduced trial demonstrated a similar consistent effect of empagliflozin on HF outcomes in patients with HF with reduced ejection fraction irrespective of diabetes status.^4,11^ The results of these 2 trials suggest that empagliflozin reduces the risk of major HF outcomes across the spectrum of chronic HF in patients with and without diabetes.

The magnitude of the effect of empagliflozin to slow the rate of eGFR decline, compared with placebo, was greater in patients with diabetes than in those without diabetes when on-treatment eGFR slope was used as the outcome. However, the effects on mean eGFR change from baseline until after treatment discontinuation was consistent in patients with and without diabetes.

One potential explanation for this discrepancy may be that the magnitude of the early eGFR dip varies; the first 4 weeks were excluded from the calculation of eGFR slope, but not in the analysis of baseline to withdrawal effects. However, whereas in EMPEROR-Reduced, empagliflozin nearly halved the risk of the renal composite end point as compared with placebo, in spite of empagliflozin slowing the rate of eGFR decline in the current EMPEROR-Preserved, a reduction in the prespecified renal composite end point was not observed in patients with or without diabetes. It has been proposed that the explanation behind this finding may be related to the applied definition of a “major renal event.”^16^

Empagliflozin reduced HbA1c levels only in patients with diabetes and only moderately, with a mean difference in change as compared with placebo of −0.19% at week 52. This latter result likely reflects good glycemic control in the diabetes population at baseline with an HbA1c of 7.3%. Hypoglycemic episodes occurred more frequently in patients with diabetes, whereas diabetic ketoacidosis occurred only in these patients, without differences in the 2 treatment arms. Empagliflozin caused a reduction in body weight both in patients with and without diabetes, but the magnitude of this effect was greater in patients with diabetes, which may be explained by the more profound glucose and calorie loss expected in these patients. In contrast, the effect of the drug on uric acid reduction was less profound in patients with diabetes than in those without diabetes. This finding does not support the hypothesis that the empagliflozin-induced reduction in uric acid is caused by increased excretion, because this excretion is linked to glucosuria, which is greater in patients with hyperglycemia.

Although prespecified‚ this study is a secondary analysis of a randomized trial and as such its results should be interpreted with caution.

In patients with symptomatic HF and an ejection fraction >40%, empagliflozin improved HF outcomes in both patients with and without diabetes. Empagliflozin provides consistent benefits across the spectrum of chronic HF and regardless of the presence of diabetes.

## Article Information

### Acknowledgments

The authors were responsible for all content and editorial decisions, were involved at all stages of development, and approved the final version. Drs Filippatos, Farmakis, and Ofstad drafted the manuscript. S. Schnaidt performed statistical analyses. All authors reviewed and edited the manuscript critically and approved the final version. Graphical assistance was provided by 7.4 Limited.

### Sources of Funding

EMPEROR-Preserved (Empagliflozin Outcome Trial in Patients With Chronic Heart Failure With Preserved Ejection Fraction) was funded by the Boehringer Ingelheim and Eli Lilly and Company Diabetes Alliance. Graphical assistance was supported financially by Boehringer Ingelheim.

### Disclosures

Dr Filippatos reports lecture fees and/or committee member contributions in clinical trials sponsored by Bayer, Medtronic, Vifor, Servier, Novartis, Amgen, and Boehringer Ingelheim and research support from the European Union. Dr Butler reports consulting fees from Bi, Cardior, Cvrx, Foundry, G3 Pharma, Imbria, Impulse Dynamics, Innolife, Janssen, LivaNova, Luitpold, Medtronic, Merck, Novartis, Novo Nordisk, Relypsa, Roche, Sanofi, Sequana Medical, V-Wave Ltd, and Vifor. Dr Farmakis reports lecture fees and/or advisory board fees from Abbott Laboratories, Bayer, Boehringer Ingelheim, Leo, Novartis, and Orion. Dr Zannad has recently received steering committee or advisory board fees from Amgen, AstraZeneca, Bayer, Boehringer Ingelheim, Boston Scientific, Cardior, Cvrx, Janssen, LivaNova, Merck, Mundipharma, Novartis, Novo Nordisk, and Vifor Fresenius. S. Schnaidt and Drs Ofstad and Brueckmann are employees of Boehringer Ingelheim. Dr Ferreira reports consultancy fees from Boehringer Ingelheim. Dr Green has received research support from Boehringer Ingelheim/Eli Lilly & Company, Merck, and Roche and is a consultant for Boehringer Ingelheim/Eli Lilly & Company, Novo Nordisk, Bayer, AstraZeneca, Sanofi/Lexicon, Pfizer, and Hawthorne Effect/Omada. Dr Rosenstock reports research funding from Applied Therapeutics Inc, Boehringer Ingelheim, Eli Lilly & Company, Genentech, GlaxoSmithKline, Hanmi, Intarcia, Janssen, Lexicon, Merck, Metacrine, Novo Nordisk, Novartis, Oramed, Pfizer, and Sanofi and advisory board consultancy fees and honoraria from Applied Therapeutics Inc, Boehringer Ingelheim, Eli Lilly & Company, Hanmi, Intarcia, Janssen, Novo Nordisk, Oramed, Sanofi, and Zealand. Dr Pocock reports consultancy fees from Boehringer Ingelheim. Dr Packer reports personal fees from Boehringer Ingelheim during the conduct of the study and personal fees from AbbVie, Actavis, Amarin, Amgen, AstraZeneca, Boehringer Ingelheim, Caladrius, Casana, Csl Behring, Cytokinetics, Imara, Lilly, Moderna, Novartis, Reata, Relypsa, and Salamandra outside the submitted work. Dr Anker reports grants and personal fees from Vifor International and Abbott Vascular and personal fees from AstraZeneca, Bayer, Brahms, Boehringer Ingelheim, Cardiac Dimensions, Novartis, Occlutech, Servier, and Vifor Int.

### Supplemental Material

Figures S1–S5

Tables S1 and S2

## Supplementary Material


