# Supplementary material for: Empagliflozin for Heart Failure With Preserved Left Ventricular Ejection Fraction With and Without Diabetes
Source: Circulation. 2022 Jun 28;146(9):676–86. doi: 10.1161/CIRCULATIONAHA.122.059785 (PMC9422757; doi:10.1161/CIRCULATIONAHA.122.059785)
Supplement: Supplementary file 1 [file cir-146-676-s001.pdf]

Supplemental Material

Table S1. Baseline Characteristics According to Glycemic Status

	**Diabetes (n=2938)**	**Pre-diabetes (n=1980)**	**Normoglycemic (n=1070)**
Age, y	70.9±9.0	73.3±9.3	72.0±10.2
Female, n (%)	1256 (42.8)	965 (48.7)	455 (42.5)
Race, n (%)*			
White	2208 (75.2)	1505 (76.0)	829 (77.5)
Black/African American	141 (4.8)	79 (4.0)	38 (3.6)
Asian	393 (13.4)	282 (14.2)	149 (13.9)
Other, including mixed race	195 (6.6)	114 (5.8)	53 (5.0)
Missing	1 (<0.1)	0	1 (0.1)
Geographic region, n (%)			
North America	365 (12.4)	234 (11.8)	120 (11.2)
Latin America	814 (27.7)	436 (22.0)	265 (24.8)
Europe	1259 (42.9)	946 (47.8)	484 (45.2)
Asia	303 (10.3)	245 (12.4)	138 (12.9)
Other	197 (6.7)	119 (6.0)	63 (5.9)
NYHA class, n (%)			
I	2 (0.1)	0	2 (0.2)
II	2332 (79.4)	1641 (82.9)	910 (85.0)
III	598 (20.4)	330 (16.7)	155 (14.5)
IV	6 (0.2)	9 (0.5)	3 (0.3)
Body mass index, kg/m^2	31.05±5.89	28.96±5.60	28.14±5.60
Heart rate, bpm	70.7±11.5	70.5±12.3	69.3±12.0
Systolic blood pressure, mmHg	133.3±16.0	130.3±15.0	130.6±15.3
Diastolic blood pressure, mmHg	75.4±10.6	76.2±10.3	75.9±10.8
HbA1c, %	7.26±1.50	5.91±0.24	5.34±0.24
LVEF			
Mean, %	53.9±8.7	54.7±8.8	54.6±8.7

<50, n (%)	1025 (34.9)	627 (31.7)	331 (30.9)
50–<60, n (%)	1007 (34.3)	675 (34.1)	376 (35.1)
≥60, n (%)	906 (30.8)	678 (34.2)	363 (33.9)
NT-proBNP, pg/mL	907 (485–165)	1089 (530–1863)	945 (491–1706)
Principal cause of HF, n (%)			
Ischemic	1217 (41.4)	600 (30.3)	300 (28.0)
Non-ischemic	1720 (58.5)	1380 (69.7)	770 (72.0)
Medical history, n (%)			
Hospitalization for HF in last 12 months	744 (25.3)	422 (21.3)	201 (18.8)
AF[†]	1342 (45.7)	1135 (57.3)	580 (54.2)
Hypertension	2760 (93.9)	1741 (87.9)	923 (86.3)
Coronary artery disease	1196 (40.7)	619 (31.3)	279 (26.1)
eGFR, mL/min/1.73 m^2			
Mean	59.7±20.7	60.3±18.5	63.7±19.4
<60, n (%)	1510 (51.4)	1018 (51.4)	460 (43.0)
UACR, mg/g	30.00 (9.72–136.00)	16.00 (6.19–44.0)	15.9 (6.19–40.66)
Normal (<30)	1457 (49.6)	1306 (66.0)	711 (66.4)
Microalbuminuria (30–300)	994 (33.8)	558 (28.2)	308 (28.8)
Macroalbuminuria (>300)	475 (16.2)	105 (5.3)	49 (4.6)
Device therapy, n (%)			
Implantable cardioverter-defibrillator[‡]	109 (3.7)	79 (4.0)	44 (4.1)
HF medication, n (%)			
ACE inhibitor	1180 (40.2)	807 (40.8)	422 (39.4)
ARB[§]	1223 (41.6)	715 (36.1)	378 (35.3)
ARNi	63 (2.1)	46 (2.3)	25 (2.3)
Diuretic other than MRA	2451 (83.4)	1588 (80.2)	770 (72.0)
MRA	1148 (39.1)	730 (36.9)	366 (34.2)

Beta blocker	2582 (87.9)	1695 (85.6)	890 (83.2)
Cardiovascular medication, n (%)			
Lipid lowering	2316 (78.8)	1306 (66.0)	620 (57.9)
Aspirin	1452 (49.4)	698 (35.3)	362 (33.8)
Anticoagulants	1268 (43.2)	1081 (54.6)	563 (52.6)

Data are mean (SD) or n (%) except for NT-proBNP and UACR that are median (IQR). ACE indicates angiotensin-converting enzyme; AF, atrial fibrillation; ARB, angiotensin receptor blocker; ARNi, angiotensin receptor neprilysin inhibitor; DPP-4, dipeptidyl peptidase-4; ECG, electrocardiogram; GFR, glomerular filtration rate; GLP-1, glucagon-like peptide-1; HbA1c, hemoglobin A1c; HF, heart failure; IQR, interquartile range; LVEF, left ventricular ejection fraction; MRA, mineralocorticoid receptor antagonist; NT-proBNP, N-terminal prohormone B-type natriuretic peptide; NYHA, New York Heart Association; SD, standard deviation; UACR, urinary-albumin creatinine ratio; and y, year.

*Race was reported by the patient; patients who identified with no race were classified as other.

†AF reported in any ECG before treatment intake or history of AF reported as medical history.

‡Implantable cardioverter-defibrillator with or without cardiac resynchronization therapy.

§Excluding valsartan when taken with sacubitril as sacubitril/valsartan is shown as an ARNi.

Table S2. Hypoglycemic AEs and Diabetic Ketoacidosis According to Treatment Arm and Glycemic Status

	**Empagliflozin (n=2996) n/N (%)**	**Placebo (n=2989) n/N (%)**
Patients with confirmed hypoglycemic episode*		
Normoglycemia	6/530 (1.1)	5/539 (0.9)
Pre-diabetes	4/1001 (0.4)	7/979 (0.7)
Diabetes	63/1465 (4.3)	66/1471 (4.5)
Patients with severe hypoglycemic episodes†		
Normoglycemia	1/530 (0.2)	0/539
Pre-diabetes	1/1001 (0.1)	2/979 (0.2)
Diabetes	22/1465 (1.5)	18/1471 (1.2)
Diabetic ketoacidosis		
Normoglycemia	0/530	0/539
Pre-diabetes	0/1001	0/979
Diabetes	4/1460 (0.3)	5/1466 (0.3)

AE indicates adverse event.

*Defined as hypoglycemic AEs with a plasma glucose value ≤70 mg/dL or that required assistance.

†Defined as hypoglycemic episodes requiring assistance.

	Empagliflozin (N=2997)			Placebo (N=2991)				Interaction
	n/N	%	Rate/100 PY	n/N	%	Rate/100 PY	HR (95% CI)	*p*-value**
**Primary outcome: Time to first event of CV death or HHF**								
All patients	415/2997	13.8	6.86	511/2991	17.1	8.67	0.79 (0.69, 0.90)	
Normoglycemia	50/530	9.4	4.49	82/540	15.2	7.59	0.60 (0.42, 0.85)	0.37
Prediabetes	126/1001	12.6	6.14	138/979	14.1	7.00	0.89 (0.70, 1.13)	
Diabetes	239/1466	16.3	8.30	291/1472	19.8	10.25	0.79 (0.67, 0.94)	
**Key secondary outcome: First and recurrent HHF^**								
All patients	407/2997	–	6.46	541/2991	–	8.60	0.73 (0.61, 0.88)	
Normoglycemia	47/530	–	4.14	77/540	–	6.83	0.59 (0.37, 0.96)	0.62
Prediabetes	110/1001	–	5.20	129/979	–	6.24	0.83 (0.59, 1.16)	
Diabetes	250/1466	–	8.19	335/1472	–	10.82	0.73 (0.57, 0.94)	
**Secondary outcomes**								
**Time to first HHF**								
All patients	259/2997	8.6	4.28	352/2991	11.8	5.97	0.71 (0.60, 0.83)	
Normoglycemia	28/530	5.3	2.52	50/540	9.3	4.63	0.56 (0.35, 0.89)	0.82
Prediabetes	76/1001	7.6	3.70	87/979	8.9	4.41	0.85 (0.62, 1.15)	
Diabetes	155/1466	10.6	5.38	215/1472	14.6	7.57	0.69 (0.56, 0.85)	
**Time to CV death**								
All patients	219/2997	7.3	3.42	244/2991	8.2	3.81	0.91 (0.76, 1.09)	
Normoglycemia	34/530	6.4	2.96	49/540	9.1	4.29	0.69 (0.45, 1.07)	0.17
Prediabetes	65/1001	6.5	3.03	72/979	7.4	3.44	0.91 (0.65, 1.27)	
Diabetes	120/1466	8.2	3.87	123/1472	8.4	3.89	0.99 (0.77, 1.27)	
**Time to all-cause mortality**								
All patients	422/2997	14.1	6.60	427/2991	14.3	6.67	1.00 (0.87, 1.15)	
Normoglycemia	67/530	12.6	5.84	80/540	14.8	7.01	0.84 (0.60, 1.16)	0.25
Prediabetes	121/1001	12.1	5.63	121/979	12.4	5.77	1.02 (0.79, 1.31)	
Diabetes	234/1466	16.0	7.54	226/1472	15.4	7.14	1.05 (0.88, 1.26)	

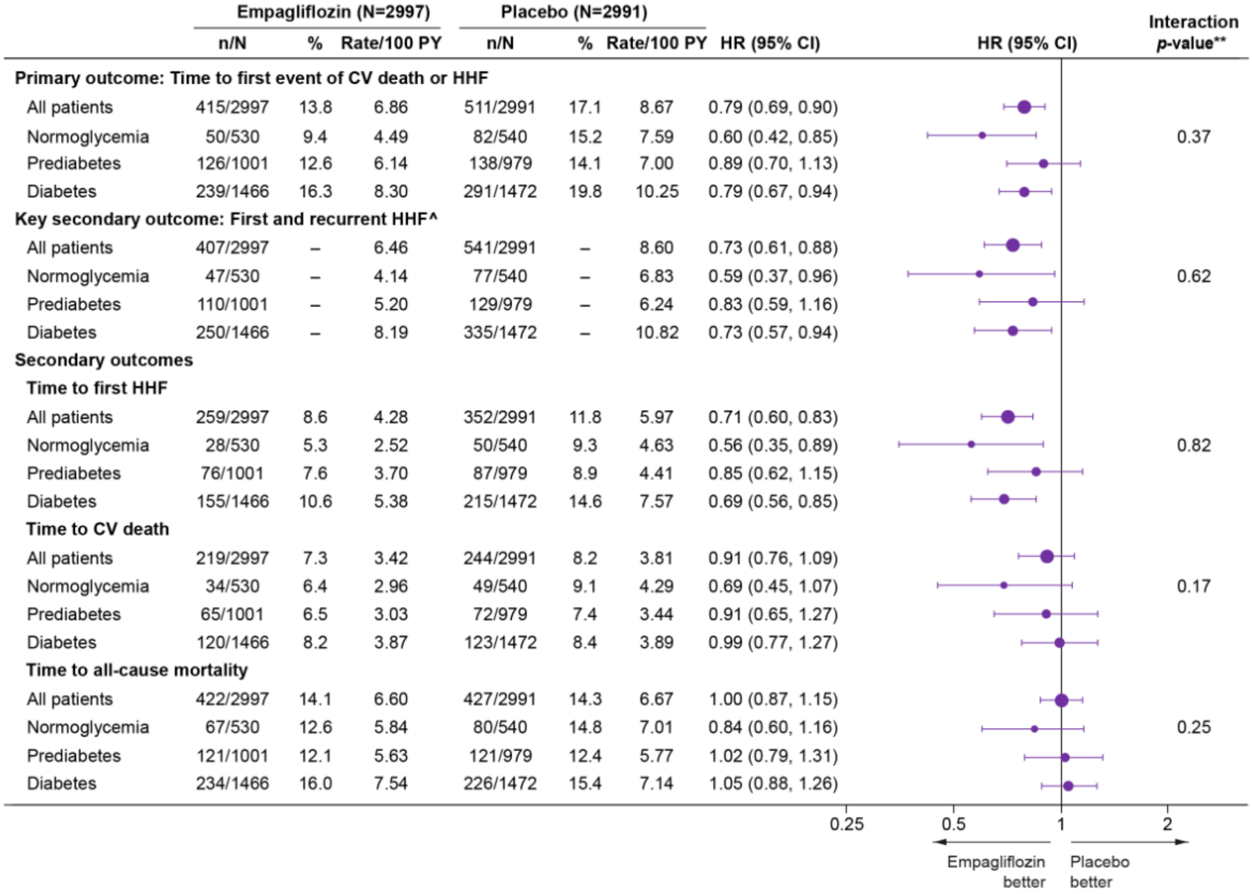

Figure S1. Forest plots for the effects of empagliflozin versus placebo on the primary endpoint and secondary cardiovascular endpoints according to glycemic status at baseline. n corresponds to the number of events in recurrent event analyses and the number of patients with events for time-to-first event analysis. ^Recurrent event analyses are based on the joint frailty model accounting for competing risk of CV death. ***P* value for treatment-by-subgroup interaction trend test. CI indicates confidence interval; CV, cardiovascular; HHF, hospitalization for heart failure; HR, hazard ratio; and PY, patient-years.

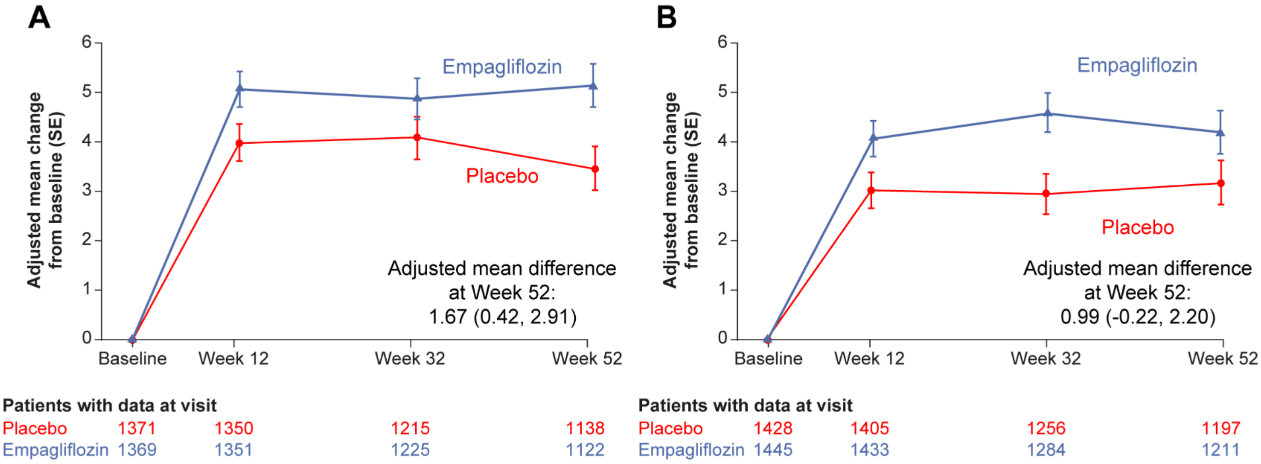

Figure S2. Adjusted mean change from baseline in KCCQ-CSS from baseline to week 52 by treatment (empagliflozin or placebo) in patients with diabetes at baseline (A), and without diabetes (B). Change from baseline in KCCQ-CSS was analyzed with a mixed model for repeated measures. The analysis is based on on-treatment data. CKD-EPI indicates Chronic Kidney Disease-Epidemiology Collaboration; eGFR, estimated glomerular filtration rate; KCCQ-CSS, Kansas City Cardiomyopathy Questionnaire-Clinical Summary Score; and SE, standard error.

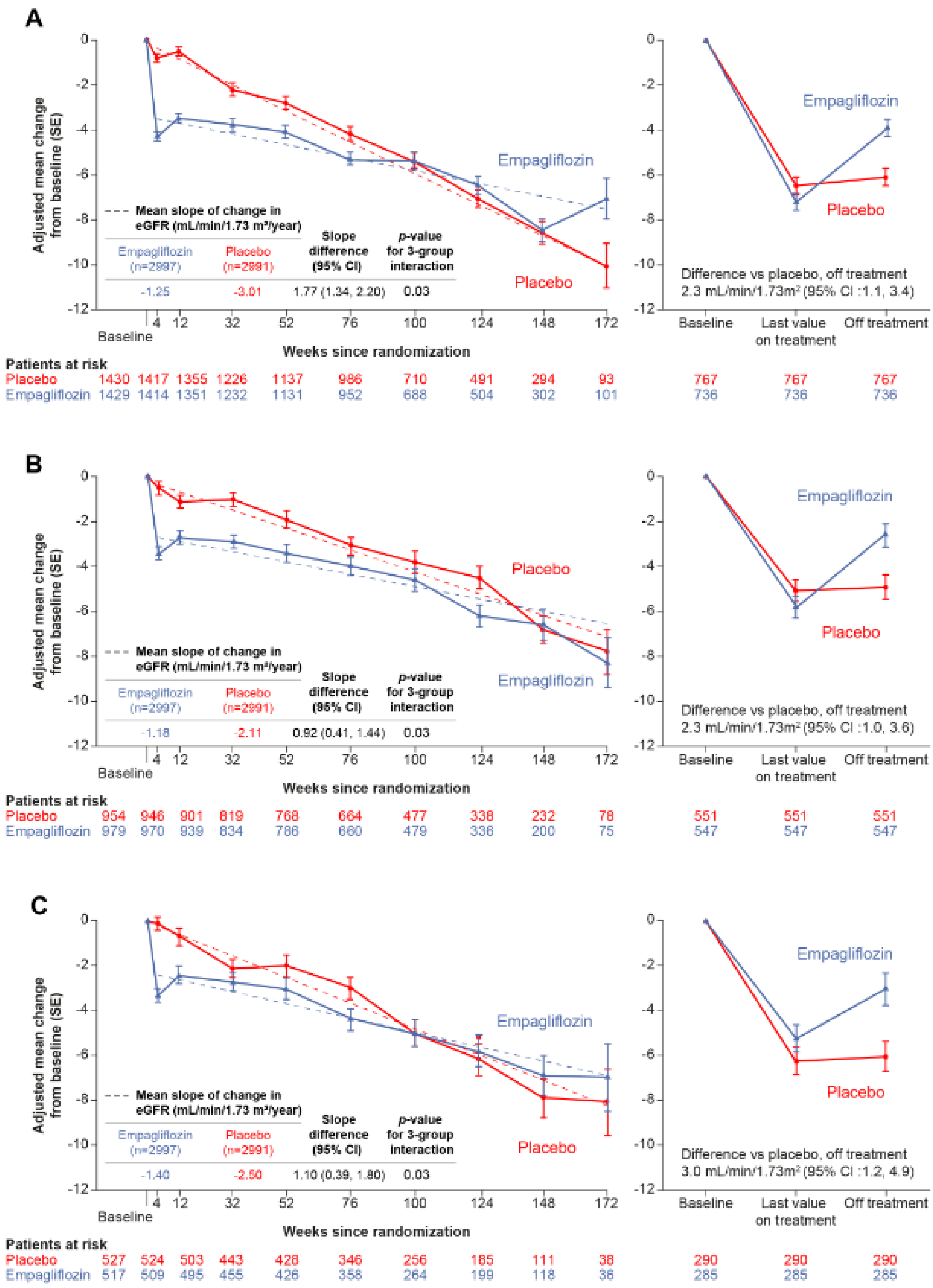

Figure S3. Adjusted mean changes from baseline in eGFR (CKD-EPI), mean slope of change in eGFR, and change in eGFR to follow-up after treatment discontinuation in patients with

diabetes (A), prediabetes (B), and normoglycemia (C). CI indicates confidence interval; CKD-EPI, the Chronic Kidney Disease Epidemiology Collaboration; eGFR, estimated glomerular filtration rate; and SE, standard error.

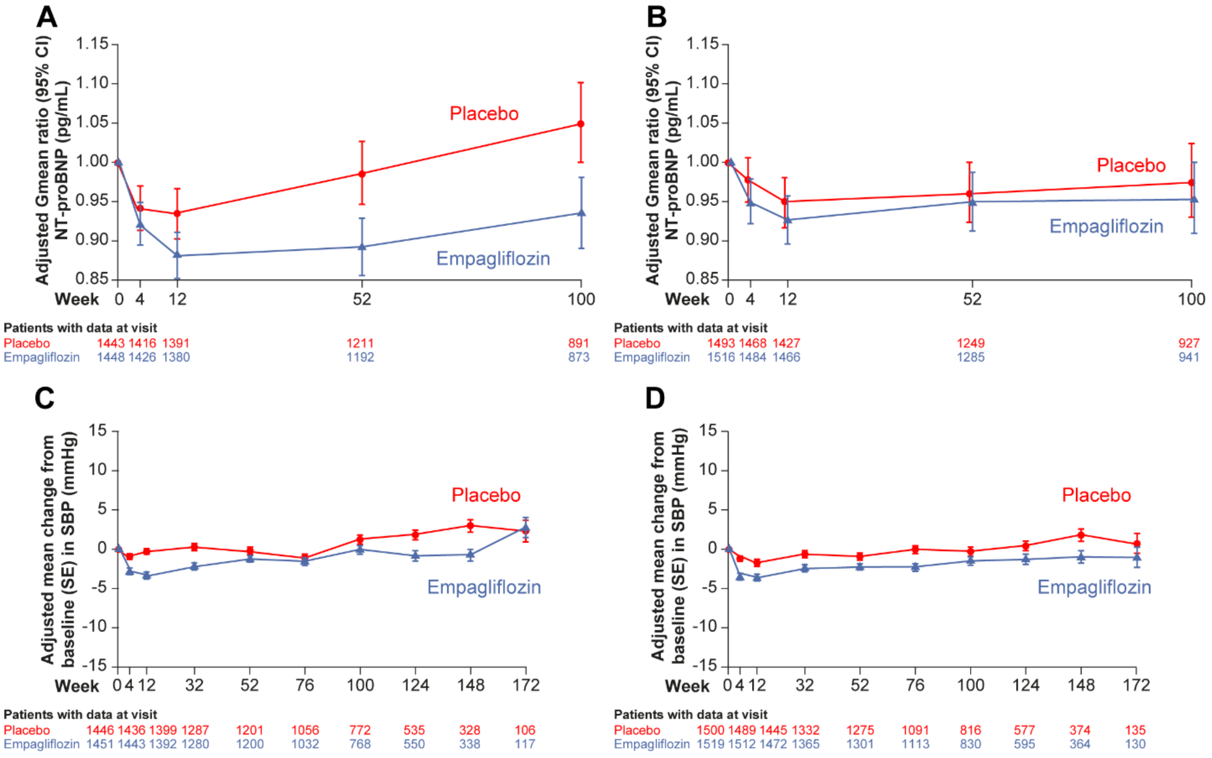

Figure S4. Change in NT-proBNP in patients with and without diabetes at baseline (A and B) and change in SBP in patients with and without diabetes at baseline (C and D). CI indicates confidence interval; NT-proBNP, N-terminal B-type natriuretic pro-peptide; SBP, systolic blood pressure; Gmean ratio, geometric mean ratio; and SE, standard error.

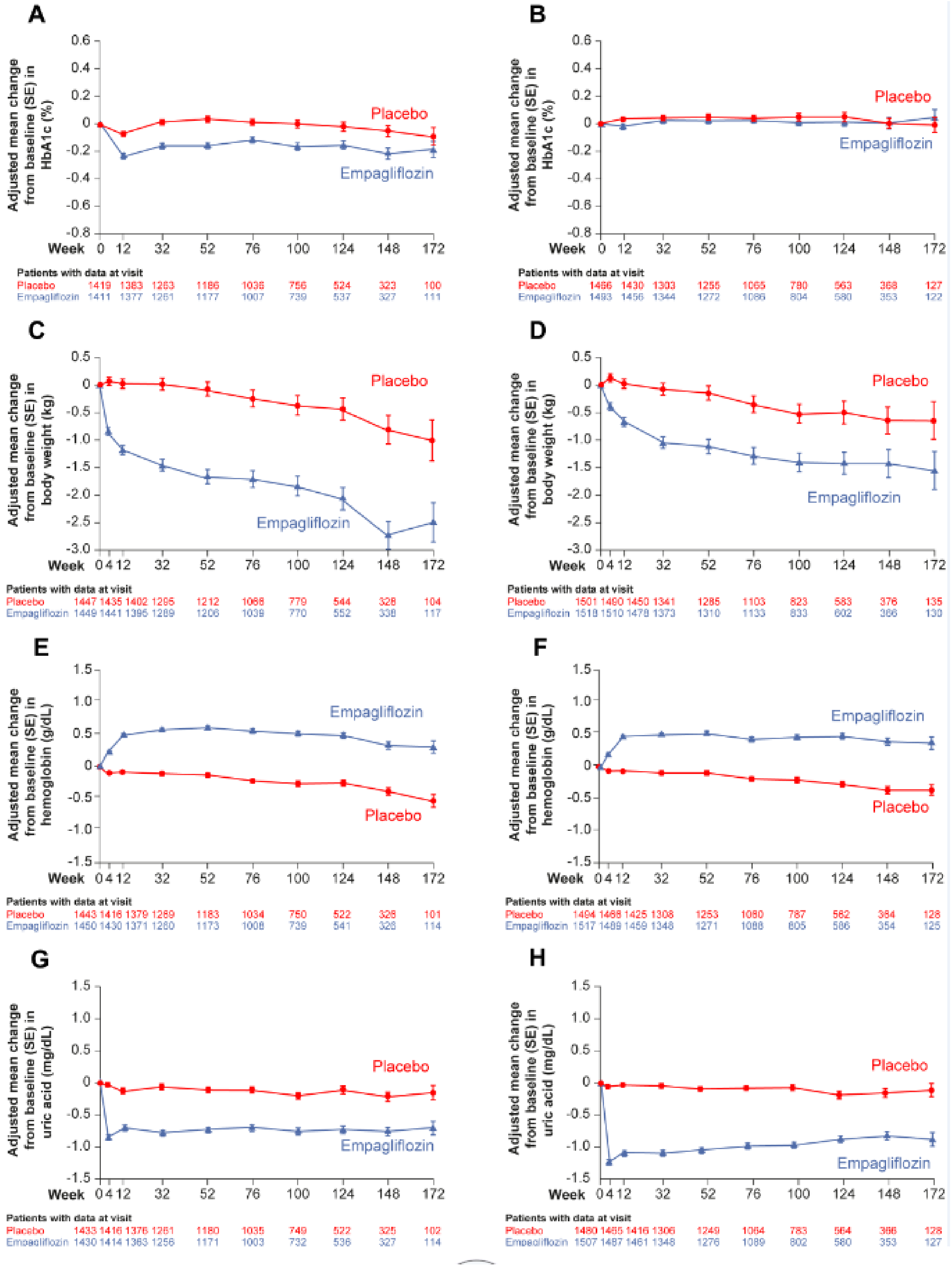

Figure S5. Adjusted mean change from baseline in body weight and laboratory outcomes in patients with and without diabetes at baseline. HbA1c in patients **A,** with diabetes, and **B,** without diabetes. Body weight in patients **C,** with diabetes, and **D,** without diabetes.

Hemoglobin in patients **E,** with diabetes, and **F,** without diabetes. Uric acid in patients **G,** with diabetes, and **H,** without diabetes. HbA1c indicates hemoglobin A1c; and SE, standard error.
